# Protocol for a cohort study: evaluating the effect of neoadjuvant immunochemotherapy on intraoperative remifentanil consumption during IoC2-guided anesthesia in elderly patients with esophageal cancer

**DOI:** 10.1080/07853890.2026.2707864

**Published:** 2026-07-29

**Authors:** Halisa Paerhati, Xinyang Hu, Yuchen Lu, Lingxi Xing, Yuyan Ding, Minhao Zhang, Lianbing Gu

**Affiliations:** ^a^ Department of Anesthesiology, the Affiliated Cancer Hospital of Nanjing Medical University, Jiangsu Cancer Hospital, and Jiangsu Institute of Cancer Research, Nanjing, China; ^b^ School of Anesthesiology, Xuzhou Medical University, Xuzhou, China

**Keywords:** Esophageal cancer, neoadjuvant immunochemotherapy, remifentanil, IoC2, opioids

## Abstract

**Background:**

The influence of neoadjuvant immunochemotherapy (nICT) on intraoperative opioid consumption during esophagectomy for cancer is not well understood. IoC2 enables real-time antinociceptive depth monitoring. This study assesses nICT’s effect on remifentanil requirements in elderly patients using IoC2.

**Methods:**

This prospective observational cohort study will be conducted at Jiangsu Cancer Hospital. Sixty patients scheduled for esophagectomy will be enrolled. Patients will be divided into the nICT group (*n* = 30) or the treatment-naive group (*n* = 30). All patients will receive a standardized anesthetic protocol. The primary outcome is the mean intraoperative remifentanil infusion rate (μg/kg/min). Secondary outcomes include: total intraoperative dose of sufentanil; postoperative cumulative opioid consumption at PACU, 12, 24, 48, and 72 h; pain scores, PCIA attempts, and rescue analgesia use at PACU, 12, 24, 48, and 72 h post-surgery, and the incidence of moderate-to-severe pain (NRS ≥ 4) at these time points; as well as delirium, postoperative complications, chest tube removal time, and hospital length of stay.

**Discussion:**

This study aims to clarify the effect of neoadjuvant therapy on opioid requirements during esophageal cancer surgery. By employing precise IoC2 monitoring, we aim to establish a methodological basis for developing individualized, precise anesthesia-analgesia strategies and to provide prospective evidence for their clinical application.

**Trial registration:**

The study protocol is registered with the Chinese Clinical Trial Registry under registration number ChiCTR2500105958, dated July 15, 2025.

## Introduction

### Esophageal cancer

Esophageal cancer is one of the most lethal malignancies worldwide. According to 2022 statistics, there were approximately 511,000 new cases globally, ranking it 11th in incidence among all cancers, and about 445,000 deaths, making it the 7th leading cause of cancer-related mortality. The incidence of esophageal cancer exhibits significant geographical variation, with high rates observed in East Asia and Eastern Africa. In China, the same year, new cases and deaths from esophageal cancer accounted for 4.64% and 7.28% of all cancer cases, respectively, with an age-standardized incidence rate (ASIR) of 15.87 per 100,000. The predominant pathological type is squamous cell carcinoma (accounting for approximately 95%). Globally, the ASIR increases significantly with age, reaching 36.50 per 100,000 in individuals aged 65 and above [1–3].

### Neoadjuvant therapy and adverse effects

For locally advanced esophageal cancer, a comprehensive treatment model combining perioperative therapy with surgery holds a crucial position. Both the National Comprehensive Cancer Network (NCCN) guidelines and the Chinese Society of Clinical Oncology (CSCO) guidelines strongly recommend neoadjuvant therapy followed by surgery as the standard treatment for locally advanced disease. Most patients are diagnosed at a locally advanced stage, typically defined as tumor invasion into the deep layers of the esophageal wall or adjacent structures, or with regional lymph node metastasis, but without distant metastasis (staged as (T3-4 anyN or anyT N+) and M0 according to the American Joint Committee on Cancer [AJCC]) [4]. For these patients, treatment with surgery alone is associated with a poor overall prognosis, with 5-year survival rates generally below 30% [5].

Neoadjuvant therapy, as a standard preoperative strategy for locally advanced resectable esophageal cancer, has been demonstrated by numerous clinical studies to offer multiple advantages: it effectively reduces clinical tumor stage, eliminates micro-metastases, increases the R0 resection rate, and improves long-term survival. Furthermore, it can render some initially unresectable or borderline resectable tumors operable. Common neoadjuvant modalities include chemotherapy, radiotherapy, immunotherapy, and targeted therapy. In recent years, with advances in immunotherapy, neoadjuvant immunotherapy combined with chemotherapy (nICT) has shown remarkable efficacy in esophageal cancer. Several phase II-III clinical trials have indicated that compared to chemotherapy alone, nICT significantly improves the pathological complete response (pCR) rate and major pathological response (MPR) rate, consequently enhancing postoperative event-free survival (EFS). Therefore, nICT has gradually become a first-line recommended regimen for these patients and has been incorporated into authoritative national and international guidelines [6,7].

However, alongside its enhanced efficacy, nICT is associated with a series of non-negligible treatment-related adverse reactions. Common adverse effects of immune checkpoint inhibitors include fatigue, rash, diarrhea, immune-mediated hepatitis (manifesting as elevated transaminases), and immune-mediated colitis, among others. Severe, potentially life-threatening immune-related adverse events such as myocarditis, pneumonitis, or encephalitis can occur. Chemotherapy agents frequently cause classic toxicities like nausea, vomiting, myelosuppression, and hepatic or renal impairment [8,9].

These treatment-related organ function impairments and worsened nutritional status may further affect a patient’s tolerance to anesthesia, including intraoperative hemodynamic stability and the pharmacodynamics of anesthetic agents, particularly the metabolism and response to opioids. Both under- and overdosing of opioids can directly impact the quality of postoperative recovery. Notably, a recent clinical observational study in esophageal cancer patients suggested that total perioperative fentanyl consumption was significantly higher in those who received nICT compared to those receiving neoadjuvant chemotherapy alone [10]. A potential mechanism underlying the increased opioid requirement in the nICT group may involve the blockade of the PD-1/PD-L1 signaling pathway by immune checkpoint inhibitors. This pathway is physiologically involved in endogenous pain signal modulation, and its pharmacological inhibition might lead to a decreased pain threshold, thereby elevating analgesic demand [11]. On the other hand, the contribution of chemotherapy-induced peripheral neuropathy (CIPN) to intraoperative opioid needs remains complex. Neuropathy caused by agents such as paclitaxel and platinum, often manifesting as paresthesia or hyperalgesia, has shown conflicting associations with intraoperative opioid requirements in the literature [12]. There is currently no conclusive evidence that chemotherapy universally increases intraoperative opioid demand. Conversely, some studies indicate that ovarian cancer patients receiving neoadjuvant chemotherapy require less PCIA postoperatively, and the severity of CIPN was negatively correlated with postoperative PCIA opioid consumption [13]. This suggests that different tumor types, chemotherapy regimens, and neuropathy characteristics may exert complex and even opposing influences on pain perception and opioid requirements, the specific mechanisms of which require further investigation.

### Index of consciousness (IoC) technology

Pain is a core perioperative stressor, the management of which relies on potent opioids like remifentanil. This agent is widely used for continuous intraoperative infusion due to its rapid onset, swift metabolism, and lack of accumulation [14]. However, traditional analgesic protocols often rely on clinical experience or weight-standardized dosing, overlooking inter-individual differences in pain sensitivity. This is particularly problematic in elderly patients, increasing the risk of respiratory depression, hemodynamic fluctuations, and postoperative delirium [15]. Therefore, developing individualized, real-time, and objective monitoring tools for analgesia is of great significance.

Traditional monitors like the Bispectral Index (BIS) primarily reflect the depth of sedation and cannot accurately assess the response to nociceptive stimuli, leading to risks of under-analgesia or opioid overdose. The recently introduced Index of Consciousness (IoC) monitoring technology, based on electroencephalogram (EEG) signals, simultaneously generates two core indices: IoC1 (also marketed as qCON, quantifying consciousness level) and IoC2 (also marketed as qNOX, nociception response index). The former quantifies sedation depth, while the latter assesses the level of analgesia. Their combined use provides a critical basis for individualized and precise anesthesia management [16]. Clinical studies have shown that IoC2-guided analgesic strategies can significantly reduce remifentanil consumption, lower the incidence of intraoperative body movement, and decrease the risk of chronic postoperative pain [17].

It remains unclear whether nICT enhances nociception through the synergistic effects of immunotherapy and chemotherapy, leading to increased intraoperative analgesic demands. Whether this phenomenon is more pronounced in elderly patients also warrants clarification. Therefore, exploring the pathophysiological mechanisms underlying altered opioid requirements in esophageal cancer patients receiving nICT under IoC2 monitoring and developing targeted intervention strategies is crucial for optimizing perioperative pain management and improving patient outcomes.

Consequently, we hypothesize that elderly esophageal cancer patients who have received nICT require a higher intraoperative dose of remifentanil to maintain IoC2 within the target analgesic range.

## Methods

### Study design and setting

**Figure 1.**
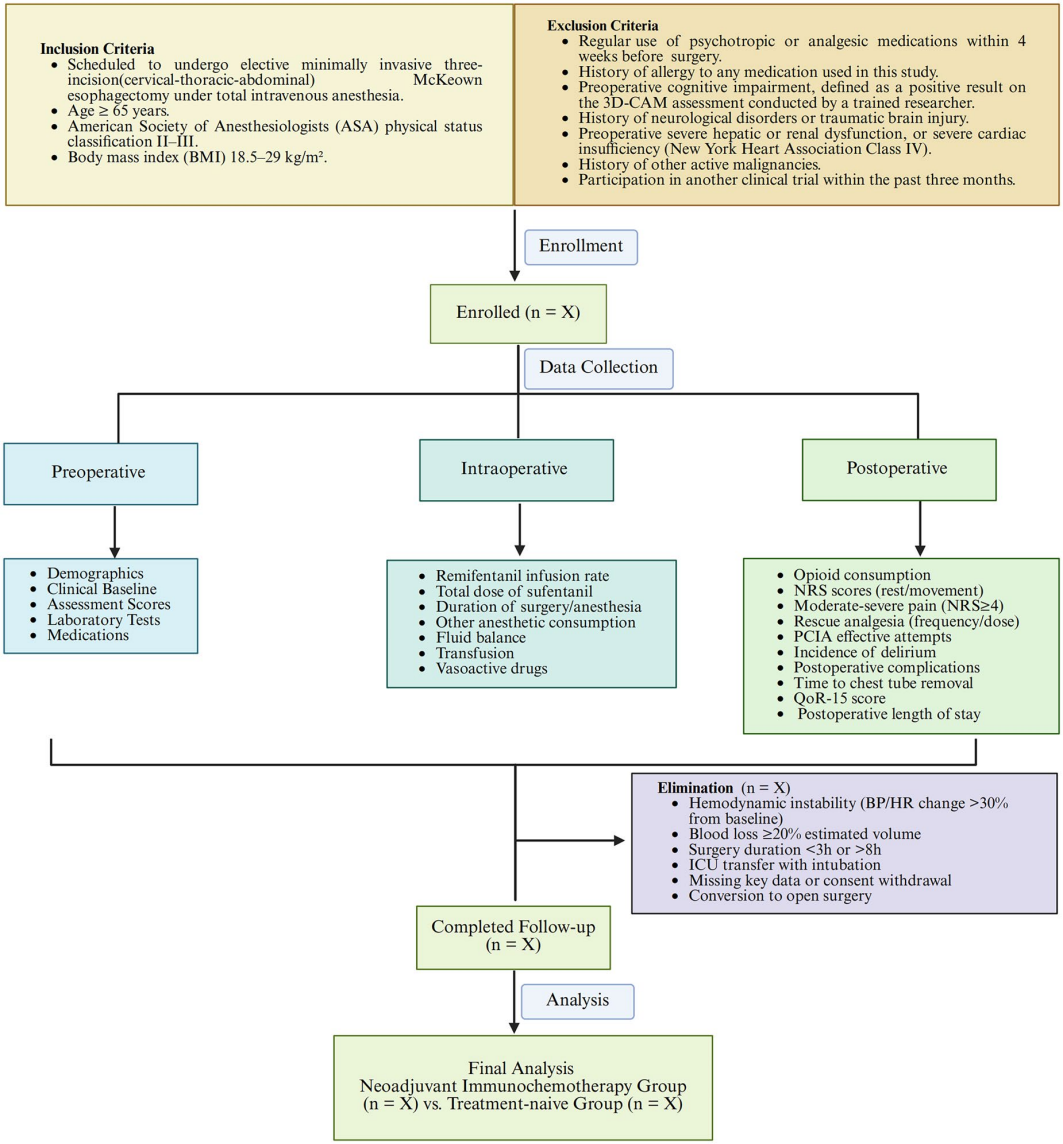
Study flow diagram.

This is a single-center, prospective, observational cohort study conducted at Jiangsu Cancer Hospital. The protocol was approved by the Ethics Committee of Jiangsu Cancer Hospital (Approval No. KY-2025-068). The study will be conducted in accordance with the principles of the Declaration of Helsinki, and all enrolled patients will be required to sign a written informed consent form. This study protocol has been written and reported in line with the SPIRIT (Standard Protocol Items: Recommendations for Interventional Trials) guidelines. The completed SPIRIT checklist has been submitted as supplementary material [18]. The study flow diagram is presented in Figure 1.

### Participants and recruitment

Jiangsu Cancer Hospital is a tertiary Grade-A teaching hospital with advanced expertise in thoracic surgery and a substantial volume of esophageal cancer cases, which provides a robust foundation for conducting this study focusing on elderly patients with esophageal cancer. A total of 60 patients are planned to be enrolled, with recruitment scheduled from 30 October 2025 to 30 June 2026.

### Recruitment process

Potential participants will initially be identified by the attending surgical team during preoperative outpatient visits or hospital admission. Subsequently, an independent researcher, not involved in clinical management or outcome assessment, will screen the electronic medical records of these patients against the inclusion and exclusion criteria. Eligible patients will be approached by this researcher, who will elaborate on the study details, explain potential risks and benefits, and invite them to participate. Written informed consent will be obtained from all willing patients before any study-related procedures are initiated.

### Inclusion criteria

Patients eligible for this study must meet all of the following criteria:

1. Scheduled to undergo elective minimally invasive three-incision (cervical-thoracic-abdominal) McKeown esophagectomy under total intravenous anesthesia.
2. Age ≥ 65 years.
3. American Society of Anesthesiologists (ASA) physical status classification II–III.
4. Body mass index (BMI) 18.5–29 kg/m^2^.

### Exclusion criteria

Patients will be excluded if they meet any of the following criteria:

1. Regular use of psychotropic or analgesic medications within 4 weeks before surgery.
2. History of allergy to any medication used in this study.
3. Preoperative cognitive impairment, defined as a positive result on the 3D-CAM (3-Minute Diagnostic Interview for CAM-defined Delirium) assessment conducted by a trained researcher.
4. History of neurological disorders or traumatic brain injury.
5. Preoperative severe hepatic or renal dysfunction, or severe cardiac insufficiency (New York Heart Association Class IV).
6. History of other active malignancies.
7. Participation in another clinical trial within the past three months.

### Group assignment and blinding

Patients will be assigned to either the neoadjuvant immunochemotherapy group (Group N, *n* = 30) or the treatment-naive group (Group TN, *n* = 30) based on whether they received neoadjuvant immunochemotherapy before surgery. Group TN consists of patients who have not received any form of neoadjuvant therapy (chemotherapy, radiotherapy, immunotherapy, or targeted therapy). For patients in Group N, a uniform neoadjuvant regimen is required: at least two cycles of a PD-1/PD-L1 inhibitor combined with taxane- and platinum-based chemotherapy(e.g. pembrolizumab or nivolumab + paclitaxel + cisplatin/carboplatin). This standardization minimizes heterogeneity arising from different chemotherapy agents, which may differentially affect pain pathways. In an observational cohort study, blinding patients and treating physicians is not feasible given the nature of the treatment. However, to minimize measurement bias, the researchers responsible for collecting all postoperative outcome data and the statistician performing the analyses will be blinded to group allocation. Outcome assessors will not have access to the oncology treatment modules in the electronic medical record system and will use dedicated case report forms (CRFs) that do not contain group information. The schedule for patient enrollment and outcome assessment is presented in Table 1.

**Table 1.** Schedule of patient enrolment and outcome assessment.

Timepoint	Study period								
	Enrolment	Allocation	Post-allocation						Close-out
	7 days pre-op	1 day pre-op	Intraop	PACU	12h post-op	24h post-op	48h post-op	72h post-op	Hospital discharge
*Patient enrolment*									
Eligibility screening	x								
Informed consent	x								
*Baseline Data*									
Demographics	x								
Medical history	x								
PG-SGA score	x								
FFP	x								
Laboratory tests/investigations		x							
*Outcome Assessment*									
*Primary Outcome*									
Remifentanil infusion rate (μg/kg/min)			x						
*Secondary Outcomes*									
Total dose of sufentanil			x						
Opioid consumption (MME)				x	x	x	x	x	
NRS at rest/movement				x	x	x	x	x	
Incidence of moderate-severe pain (NRS ≥ 4)				x	x	x	x	x	
3D-CAM delirium assessment	x					x	x	x	
Rescue analgesia (episodes & dose)				x	x	x	x	x	
PCIA effective attempts				x	x	x	x	x	
*Intraoperative Adverse Events*									
Hypotension			x	x					
Hypertension			x	x					
Bradycardia			x	x					
Tachycardia			x	x					
Hypoxemia (SpO₂ <90%)			x	x					
*Postoperative Recovery*									
Postoperative complications						x	x	x	
Time to chest tube removal									x
Quality of recovery-15 (QoR-15) score									x
Postoperative length of stay									x

PG-SGA: Patient-Generated Subjective Global Assessment; FFP: Fried Frailty Phenotype (FFP); MME: morphine milligram equivalents; NRS: Numerical Rating Scale pain score (0 = no pain, 10 = worst imaginable pain); PCIA:patient-controlled intravenous analgesia; 3D-CAM: 3-Minute Diagnostic Interview for CAM-defined Delirium; PACU: post-anaesthesia care unit.

### Anesthesia

#### Preparation and monitoring

Patients were required to fast for 8 h and abstain from clear fluids for 4 h preoperatively. Upon entering the operating room, baseline blood pressure (BP), heart rate (HR), and pulse oximetry (SpO**_2_**) were measured and recorded. Radial artery cannulation was performed under local anesthesia for continuous invasive arterial pressure monitoring. Intraoperative monitoring included continuous electrocardiography (ECG), invasive arterial pressure, SpO**_2_**, end-tidal carbon dioxide (ETCO**_2_**), and the Consciousness Index (IoC1 and IoC2, equipment provided by Shenzhen Well Health Medical Technology Co., Ltd.).

#### Anesthesia induction

A standardized induction protocol was employed: intravenous administration of sufentanil (0.3 µg/kg), midazolam (0.03 mg/kg), propofol (1 mg/kg), and rocuronium (0.6 mg/kg). Tracheal intubation and placement of a bronchial blocker under fiberoptic bronchoscopic guidance were performed once neuromuscular monitoring indicated a Train-of-Four (TOF) count of 0, and both IoC1 and IoC2 values decreased below 40 and 35, respectively.

#### Anesthesia maintenance

Anesthesia was maintained using target-controlled infusion (TCI) of propofol (Marsh model, initial target concentration 4.0 µg/mL, maintenance range 3–6 µg/mL) and remifentanil (Minto model, initial target concentration 4.0 ng/mL, maintenance range 2–6 ng/mL). Sedation Depth: IoC1 > 60 was defined as inadequate sedation, and IoC1 < 40 as excessive sedation. The target range for IoC1 was 40–60. If IoC1 remained >60 for over 1 min, the propofol target concentration was increased by 0.5 µg/mL; if it remained < 40 for over 1 min, the concentration was decreased by 0.5 µg/mL. Analgesia Depth: IoC2 > 55 was defined as inadequate analgesia, and IoC2 < 35 as excessive analgesia. The target range for IoC2 was 35–55. If IoC2 remained > 55 for over 1 min, the remifentanil target concentration was increased by 1 ng/mL; if it remained < 35 for over 1 min, the concentration was decreased by 1 ng/mL.

#### Mechanical ventilation

During two-lung ventilation, Pressure Controlled Ventilation-Volume Guaranteed (PCV-VG) mode was used with a tidal volume of 6–8 mL/kg, PEEP of 5 cm H_2_O, and a fractional inspired oxygen concentration (FiO_2_) of 60%, aiming to maintain SpO_2_ ≥ 95% and ETCO_2_ between 35 and 45 mmHg.

During one-lung ventilation, the PCV-VG mode was maintained with a tidal volume of 4–6 mL/kg and PEEP of 5 cm H_2_O. FiO_2_ and respiratory rate were adjusted individually to maintain SpO_2_ ≥ 90% and ETCO_2_ between 35 and 45 mmHg.

#### Intraoperative management

Lactated Ringer’s solution was administered intravenously. Patient temperature was maintained at 36-37 °C (nasopharyngeal) using a warming blanket. Before chest closure, the surgeon performed an intercostal nerve block under thoracoscopic guidance at the surgical incision site and adjacent intercostal spaces using 0.25% ropivacaine (3 mL per site). Sufentanil (0.2 µg/kg) was administered intravenously 30 min before the end of surgery as an analgesic bridge. All patients received intravenous ondansetron (4 mg) at the conclusion of surgery for prophylaxis against postoperative nausea and vomiting (PONV).

#### Postoperative analgesia

PCIA was employed using a solution of sufentanil (2.5 µg/kg) and dexmedetomidine (2.5 µg/kg) diluted in 100 mL of normal saline. The background infusion rate was set at 1 mL/h, with a bolus dose of 1.5 mL and a lockout interval of 15 min.

#### Emergence and extubation

Patients were transferred to the PACU after surgery. Sugammadex (2 mg/kg) was administered intravenously upon reappearance of the second twitch (T2) on neuromuscular monitoring. Tracheal extubation was performed after thorough airway suctioning, once the TOF ratio recovered to ≥ 90%, and the patient regained consciousness, adequate spontaneous respiration, and protective airway reflexes (cough, swallow). The modified Aldrete score was assessed every 5 min post-extubation; patients with a score ≥ 9 were eligible for discharge from PACU.

#### Rescue analgesia

If the Numerical Rating Scale (NRS) pain score was ≥ 4 in the PACU or ward, rescue analgesia was administered: intravenous tramadol (50–100 mg) in the PACU, or intramuscular morphine (10 mg) in the ward.

### Outcome measures

#### Primary outcome

The primary outcome of this trial is the intraoperative remifentanil infusion rate (μg/kg/min), calculated as the total dose of remifentanil administered *via* target-controlled infusion (TCI, Minto model) divided by the duration of anesthesia and the patient’s actual body weight. Remifentanil titration is guided by the IoC2 index, with a target range of 35–55. The infusion rate is continuously recorded by the TCI pump, and the total dose is extracted from the pump log at the end of surgery.

#### Secondary outcomes

Secondary outcomes include:

1. Total dose of sufentanil (μg);
2. Postoperative opioid consumption;
3. Postoperative NRS pain scores at rest and during movement;
4. Incidence of moderate-to-severe pain(NRS ≥ 4);
5. Incidence of postoperative delirium (assessed over the first three postoperative days with 3D-CAM);
6. Total number of PCIA effective attempts;
7. Frequency of rescue analgesia and dose of rescue medication;
8. Quality of postoperative recovery, including incidence of postoperative complications; time to chest tube removal; Quality of Recovery-15 (QoR-15) score; postoperative hospital length of stay.

Postoperative cumulative opioid consumption, pain Numerical Rating Scale (NRS) scores at rest and during activity, effective patient-controlled intravenous analgesia (PCIA) attempts, and frequency and dosage of rescue analgesia assessed at PACU, 12, 24, 48, and 72 h post-surgery, and the incidence of moderate-to-severe pain (NRS ≥ 4) at these time points; as well as the incidence of postoperative delirium (assessed at 24, 48, and 72 h post-surgery). Patients will be followed up daily between 16:00 and 18:00 to collect this data. All PACU assessments will use the same definitions and criteria as ward assessments.

Opioid consumption will be standardized for analysis by converting all doses to intravenous morphine equivalents: sufentanil 10 µg = morphine 10 mg, tramadol 100 mg = morphine 20 mg [19,20].

#### Safety outcomes

Safety outcomes encompass perioperative hemodynamic events and interventions administered for these events, defined as:

1. Hypotension: mean arterial pressure (MAP) < 65 mmHg, or a decrease ≥ 20% from baseline, lasting at least 1 minute (whichever criterion is met first).
2. Hypertension: MAP > 110 mmHg (or systolic blood pressure > 160 mmHg), or an increase ≥ 30% from baseline, lasting at least 1 minute (whichever criterion is met first).
3. Bradycardia: heart rate ≤ 45 beats per minute, lasting at least 1 minute.
4. Tachycardia: heart rate > 100 beats per minute, lasting at least 1 minute.

These events will be assessed during anesthesia and the PACU stay. Should any occur, they will be managed at the discretion of the attending anesthesiologist, with pharmacological options including: ephedrine (6–10 mg) or metaraminol (100–200 µg) for hypotension; atropine (0.5 mg) for bradycardia; and urapidil (5 mg) or esmolol (10 mg) for hypertension and heart rate control.

### Data collection and management

- Preoperative Data
  - Demographics: age, sex, BMI, education, smoking, and alcohol history
  - Clinical Baseline: comorbidities (hypertension, diabetes, CAD), ASA status, tumor stage, neoadjuvant therapy details (cycles, interval-to-surgery) for patients in Group N.
  - Assessment Scores: 3D-CAM, PG-SGA, Fried Frailty Phenotype (FFP)
  - Laboratory Tests: liver/renal function (ALT, AST, creatinine, urea, uric acid, albumin, A/G ratio), hematology (hemoglobin), electrolytes (K^+^, Ca^2+^, Na^+^, Cl^-^), ECG, cardiac ultrasound
  - Medications: antiemetics, antihypertensives, hypoglycemics, lipid-lowering agents, anticoagulants/antiplatelets
- Intraoperative Data
  - Duration of surgery/anesthesia
  - Anesthetic consumption: remifentanil(µg/kg/min), sufentanil (µg), propofol (mg/kg/h)
  - Fluid balance: crystalloids, colloids, urine output, estimated blood loss
  - Transfusion: plasma, PRBCs, platelets
  - Vasoactive drugs: metaraminol, ephedrine, atropine
- Postoperative Data
  - PACU Stay: opioid consumption, NRS scores (rest/movement), moderate-severe pain (NRS ≥ 4), rescue analgesia (frequency/dose), PCIA effective attempts
  - At 12, 24, 48, and 72 h postoperatively, the same data on PACU and incidence of postoperative delirium (assessed at 24, 48, and 72 h post-surgery)
  - Additional: postoperative complications, time to chest tube removal, QoR-15 score, Postoperative length of stay

All data will be sourced from the hospital’s electronic medical record system, anesthesia charts, nursing records, and validated assessment scales (e.g. 3D-CAM for delirium). Laboratory values and imaging results are obtained from standardized hospital laboratory and radiology departments. Data collection will adhere to pre-defined Standard Operating Procedures. We have established a Data Oversight Group consisting of two clinicians not involved in patient enrollment or anesthesia management, and one team member with statistical expertise in clinical data processing. This group is responsible for periodic source data verification to ensure data completeness and logical consistency. Data will be entered independently by two personnel into a secure electronic database system, which incorporates automatic range checks and logic validation. Regular random cross-checks will be performed by comparing the electronic data against source documents (medical records). All research personnel involved in data collection (including anesthesiologists, surgeons, and assessors) will receive standardized training before study initiation. Training content includes: study protocol review; standardized assessment methods for key variables (particularly the 3D-CAM, PG-SGA, and Fried Frailty Phenotype scales); data recording specifications; and the use of the electronic data capture system. Personnel must pass a standardized assessment following training before participating in data collection.

### Sample size calculation

The primary outcome is the mean intraoperative remifentanil infusion rate (µg/kg/min), calculated as the total dose of remifentanil administered *via* target-controlled infusion (TCI, Minto model) divided by the duration of anesthesia and the patient’s actual body weight. In a pilot study conducted at our center involving 9 elderly patients undergoing thoracoscopic-laparoscopic esophagectomy, the mean remifentanil infusion rate in the treatment-naive group was 0.15 ± 0.05 µg/kg/min, while in the neoadjuvant immunochemotherapy (nICT) group it was 0.22 ± 0.05 µg/kg/min, yielding an absolute difference of 0.07 µg/kg/min (a 46.7% increase). Given the small pilot sample, this estimate may be inflated. To adopt a more conservative and clinically plausible effect size, we targeted a 25% increase in remifentanil requirement in the nICT group, corresponding to an absolute difference of 0.0375 µg/kg/min (Cohen’s *d* = 0.75). This magnitude is supported by a recent multicenter prospective study [21], which demonstrated a 22% increase in perioperative opioid consumption in patients with non-small cell lung cancer receiving neoadjuvant immunochemotherapy compared to those receiving chemotherapy alone. Although the control groups differ, the consistent direction and magnitude of the effect across studies suggest that a 25% increase is both clinically meaningful and mechanistically plausible.

With a two-sided α of 0.05 and a desired power of 90%, a minimum of 29 patients per group is required to detect a difference of 0.0375 µg/kg/min with a standard deviation of 0.05 µg/kg/min (calculated using PASS (V.11.0.7, NCSS, Kaysville, UT)). Based on our pilot experience and improved perioperative management in the main study, we anticipated a low dropout rate of approximately 5%. Accounting for this, we aimed to enroll 30 patients per group, resulting in a total sample size of 60 patients. This sample size provides 90% power to detect the predefined clinically meaningful difference, even with minimal attrition.

### Statistical analysis

All statistical analyses will be performed using IBM SPSS Statistics version 26 (IBM, Armonk, NY) and GraphPad Prism 9.0 (GraphPad Software, San Diego, CA), with a two-sided *p*-value < 0.05 considered statistically significant. Continuous variables will first be tested for normality using the Shapiro-Wilk test: normally distributed variables will be presented as mean ± standard deviation (*SD*) and compared between groups using independent samples t-tests; non-normally distributed variables will be presented as median and interquartile range (IQR) and compared using the Mann-Whitney *U* test, with Hodges-Lehmann median differences and 95% confidence intervals (CIs) reported. Categorical variables will be presented as numbers (percentages) and compared using the chi-square test or Fisher’s exact test, as appropriate, based on expected frequencies.

For the primary outcome, a multivariable linear regression model will be performed to assess the independent effect of group allocation on intraoperative remifentanil consumption. Given the limited sample size (*n* = 60) and to avoid overfitting and bias associated with *P*-value-based variable selection, bivariable screening will not be used. Instead, based on clinical relevance, a limited set of key covariates potentially influencing the primary outcome will be directly entered into the model: body mass index, anesthesia duration, alcohol history, and number of neoadjuvant therapy cycles. Results will be presented as standardized coefficients (*β*) with 95% confidence intervals (CIs).

Postoperative NRS scores at different time points will be analyzed using linear mixed models, with treatment group, time point, and their interaction as fixed effects, and subject as a random intercept.

For important binary outcomes, the number of positive events will first be assessed. According to methodological recommendations, multivariable logistic regression models require at least 10–15 positive events per predictor variable to ensure stable and reliable estimates. If the event count meets this criterion, multivariable logistic regression will be performed for adjustment, with results presented as odds ratios (OR) and 95% CIs. If the event count is too low, analysis will be limited to descriptive statistics and univariable Fisher’s exact test for group comparison, without performing multivariable adjustment, to avoid unstable models that could produce misleading results.

Given the limited sample size and the exploratory nature of this study, for multiple comparisons involving secondary outcomes, we will not strictly rely on Bonferroni correction. Instead, we will primarily base our interpretation on effect sizes (mean differences for continuous variables, odds ratios for categorical variables) along with their 95% confidence intervals, integrating clinical relevance when assessing the potential value of findings. All results from secondary outcome analyses will be considered exploratory findings, intended to generate hypotheses for future research rather than to provide confirmatory evidence.

## Discussion

This study plans to enroll 60 elderly patients scheduled for total intravenous anesthesia with minimally invasive three-Incision (cervical-thoracic-abdominal) McKeown esophagectomy, aiming to investigate the impact of neoadjuvant immunochemotherapy (nICT) on intraoperative remifentanil consumption. Furthermore, it will compare differences between groups regarding total perioperative opioid consumption, postoperative pain scores, incidence of delirium, requirement for rescue analgesia, frequency of PCIA attempts, postoperative complications, Postoperative Length of Stay, safety outcomes, and other outcomes.

Previous research suggests that nICT may enhance pain sensitivity and increase opioid requirements. In a study of patients with non-small cell lung cancer, the total perioperative opioid consumption (defined as the sum of intraoperative and postoperative use within three days) was significantly higher in those receiving neoadjuvant immunochemotherapy (nICT) compared to the neoadjuvant chemotherapy-only (nCT) group, with a mean difference of 60.39 µg. The nICT group also exhibited a significantly higher incidence of moderate-to-severe pain within 72 h postoperatively (75.6% vs. 52.5%) and significantly increased PCIA attempts at 24 and 72 h [21]. This indicates that nICT may enhance patients’ pain sensitivity and elevate opioid demand. The underlying mechanisms may be related to the following three aspects:

First, nICT may induce nociceptive sensitization by triggering a systemic inflammatory response. Immunochemotherapy can activate immune cells such as T cells and macrophages, promoting the release of pro-inflammatory cytokines like tumor necrosis factor-alpha (TNF-α), interleukin-1β (IL-1β), and IL-6. Studies show that elevated IL-6 levels in prostate cancer patients undergoing chemotherapy correlate positively with pain intensity [22]. In animal experiments, blocking cytokine pathways alleviates neuropathic pain behaviors [23]. These inflammatory mediators can act on peripheral and central nociceptive pathways, lowering the pain threshold and enhancing pain signal transmission, thereby exacerbating responses to intraoperative noxious stimuli and requiring higher remifentanil doses to maintain adequate analgesia.

Second, chemotherapeutic agents themselves may cause peripheral nerve injury, further increasing pain sensitivity. Taxanes and platinum agents, common components of nICT, can impair mitochondrial function in dorsal root ganglion neurons, induce oxidative stress and DNA damage, leading to sensory axon degeneration and abnormal expression of ion channels such as TRPV1 and sodium channels, thus inducing neuropathic pain [24–26]. Such pain often manifests as spontaneous pain, hyperalgesia, and allodynia, which may translate into stronger nociceptive responses and higher opioid requirements during surgery.

Furthermore, immune checkpoint inhibitors may disrupt endogenous analgesic mechanisms *via* the PD-1/PD-L1 pathway, contributing to an imbalance in pain regulation. Under physiological conditions, PD-1 is expressed on sensory neurons and immune cells. Its binding to the ligand PD-L1 can inhibit neuronal excitability and inflammatory cytokine release, exerting an endogenous analgesic effect [27–30]. For example, PD-L1 can inhibit sodium channel activity and activate TREK2 potassium channels *via* the PD-1/SHP pathway, thereby reducing neuronal excitability [11,31]. Research indicates that PD-L1 expression is upregulated after spinal cord injury, alleviating neuroinflammation and neuropathic pain by suppressing the activation of p38 and ERK1/2 pathways and reducing pro-inflammatory factor production [32,33]. PD-1 can also interact with the μ-opioid receptor, enhancing the inhibitory effects of opioids on calcium channels and synaptic transmission, thereby potentiating their analgesic efficacy [34–36]. Tumor cells with high PD-L1 expression can inhibit the anti-tumor activity of T cells through PD-1 binding, achieving immune escape; immune checkpoint inhibitors targeting such tumors provide effective tumor control [37]. However, PD-1/PD-L1 inhibitors used in nICT block this pathway, potentially weakening pain transmission inhibition while enhancing immune activity and inflammatory release. This may overall amplify central and peripheral pain responses, leading to increased intraoperative analgesic requirements. Basic research has confirmed that exogenous PD-L1 significantly raises the pain threshold in normal mice and those with bone cancer pain [34,38,39]. The analgesic peptide H-20 can also effectively inhibit acute and chronic pain *via* the PD-1 pathway, with no significant adverse effects observed in various preclinical pain models [40,41].

All patients in this study received a standardized multimodal analgesia regimen, including intraoperative ropivacaine intercostal nerve block and postoperative sufentanil-dexmedetomidine PCIA. Intercostal nerve block can effectively anesthetize the intercostal nerves, the primary source of pain in thoracic surgery, and is widely used for postoperative analgesia in thoracic surgery [42,43]. Studies show it significantly reduces early postoperative pain scores, decreases morphine consumption, and facilitates early recovery [44]. In this protocol, PCIA was set with a low basal infusion rate combined with patient-controlled bolus doses, aiming to provide timely and effective rescue analgesia.

This study has several limitations. First, the study was designed to compare postoperative outcomes between patients receiving neoadjuvant immunochemotherapy and those receiving no neoadjuvant treatment, without a comparison group receiving chemotherapy alone or other dual-therapy regimens. This design choice precludes direct assessment of the incremental effect of immunotherapy over chemotherapy alone. To mitigate the risk of treatment-related heterogeneity, we strictly defined the control group as patients who received no neoadjuvant treatment, thereby avoiding confounding arising from mixed treatment types; however, this approach also means that comparisons between different neoadjuvant regimens could not be explored. Second, while the study focuses on short-term pain outcomes and opioid consumption within 72 h postoperatively, it does not observe or analyze long-term postoperative pain, such as chronic post-surgical pain. The incidence and duration of long-term pain are significant for assessing overall patient prognosis and quality of life. Third, this study is subject to confounding by indication, an inherent limitation of observational research. In real-world practice, the decision to administer neoadjuvant immunochemotherapy is influenced by multiple non-random factors (e.g. frailty, nutritional status, resectability judgment, socioeconomic factors) that may also affect opioid requirements. Although we collected and adjusted for key baseline variables (ASA, BMI, PG-SGA, FFP, comorbidities, laboratory parameters), residual confounding from unmeasured factors cannot be fully excluded. Therefore, our findings should be considered exploratory and hypothesis-generating. The modest sample size (*n* = 60) may also limit detection of small effect sizes. Fourth, although the incidence of postoperative delirium (POD) was assessed within 72 h postoperatively, there is a lack of follow-up on its long-term impact, such as postoperative cognitive dysfunction (POCD). POD can significantly affect patient recovery and quality of life, and long-term follow-up could provide a more comprehensive understanding of the incidence and duration of POCD.

If our hypothesis is confirmed, that elderly patients with esophageal cancer who have received neoadjuvant immunochemotherapy require higher intraoperative doses of remifentanil to maintain adequate analgesic depth, this finding would have direct implications for clinical practice. During preoperative assessment, anesthesiologists should pay particular attention to whether patients have undergone neoadjuvant immunochemotherapy, as well as the specific regimen and number of cycles, to identify early those at increased risk for higher opioid requirements. For such patients, relying solely on high-dose opioids intraoperatively may increase the risk of adverse effects such as respiratory depression and postoperative nausea and vomiting. Therefore, individualized multimodal analgesia strategies—combining nonsteroidal anti-inflammatory drugs or regional nerve blocks with opioids—should be employed to meet the increased analgesic demands while minimizing opioid-related adverse effects, thereby enhancing postoperative recovery.

## Supplementary Material

SPIRIT.pdf

## Data Availability

No datasets were generated or analysed during the current study.
